# Investigating the Relationship between Facial Mimicry and Empathy

**DOI:** 10.3390/bs12080250

**Published:** 2022-07-24

**Authors:** Yevgeniya Kovalchuk, Elizabeta Budini, Robert M. Cook, Andrew Walsh

**Affiliations:** 1 Department of Computer Science, University of Reading, Reading RG6 6DH, UK; 2 School of Computing and Digital Technology, Birmingham City University, Birmingham B5 5JU, UK; elizabeta.budini@mail.bcu.ac.uk; 3 Centre for Social Care, Health and Related Research, Birmingham City University, Birmingham B5 5JU, UK; robert.cook@bcu.ac.uk; 4 School of Nursing and Midwifery, Birmingham City University, Birmingham B5 5JU, UK; andrew.walsh@bcu.ac.uk

**Keywords:** affective computing, empathy, facial mimicry, facial recognition technology, deep learning

## Abstract

Facial expressions play a key role in interpersonal communication when it comes to negotiating our emotions and intentions, as well as interpreting those of others. Research has shown that we can connect to other people better when we exhibit signs of empathy and facial mimicry. However, the relationship between empathy and facial mimicry is still debated. Among the factors contributing to the difference in results across existing studies is the use of different instruments for measuring both empathy and facial mimicry, as well as often ignoring the differences across various demographic groups. This study first looks at the differences in the empathetic abilities of people across different demographic groups based on gender, ethnicity and age. The empathetic ability is measured based on the Empathy Quotient, capturing a balanced representation of both emotional and cognitive empathy. Using statistical and machine learning methods, this study then investigates the correlation between the empathetic ability and facial mimicry of subjects in response to images portraying different emotions displayed on a computer screen. Unlike the existing studies measuring facial mimicry using electromyography, this study employs a technology detecting facial expressions based on video capture and deep learning. This choice was made in the context of increased online communication during and after the COVID-19 pandemic. The results of this study confirm the previously reported difference in the empathetic ability between females and males. However, no significant difference in empathetic ability was found across different age and ethnic groups. Furthermore, no strong correlation was found between empathy and facial reactions to faces portraying different emotions shown on a computer screen. Overall, the results of this study can be used to inform the design of online communication technologies and tools for training empathy team leaders, educators, social and healthcare providers.

## 1. Introduction

Facial recognition technology has largely been used to study individual psychology. However, another compelling application of this technology is the understanding and enhancement of interpersonal interactions. For example, the analysis of how we react to others’ emotional states can inform the design of tools for strengthening empathetic responses and developing interpersonal communication skills and emotional intelligence. Several social settings can benefit from such tools, including team collaboration [1], education [2] and healthcare [3]. For example, patient-perceived empathy has been shown to lead to better treatment outcomes in both physical [4,5,6] and mental [7,8] healthcare provisions. Hence, a tool helping healthcare practitioners improve their empathetic ability would be useful. Empathy in this context can be defined as the ability to recognise another person’s thoughts and feelings, and to respond to these with an appropriate emotion [9]. To be able to track the improvement in someone’s ability to empathise during interaction sessions, objective and subjective measures of empathy exhibited by the sender (e.g., healthcare practitioner) and perceived by the receiver (e.g., patient) are required.

A potential objective measure of empathy, especially in the context of online communication and training, is facial mimicry. Previous studies have shown that when exposed to other people’s emotional expressions, we tend to subconsciously imitate the other person’s expression [10,11,12]. This subconscious mimicry was labelled by Chartrand and Bargh [13] as the “chameleon effect”. Their research also suggested that “dispositionally empathic individuals exhibit the chameleon effect to a greater extent than do other people”. While the relation between the ability to automatically mimic others and empathise has been previously researched [14,15,16,17], there are no conclusive results on how to map one to the other, and the factors impacting the relation between the two. For example, Wrobel et al. [17] and Peng et al. [18] suggest that the mimicry response may be mediated by perceptions of the social meaning of the expressed emotion. Both studies found that mimicry was present in relation to happiness, absent in perception of sadness, while Wrobel et al. [17] identified a mixed pattern of response when anger was expressed. At the same time, the findings reported in [19] suggest that facial mimicry is emotion-specific, rather than just valence-based. Wrobel and Imbir [20] summarise the ongoing discussion on the extent to which social appraisal of perceived facial expression may moderate the mimicry response. In particular, the authors argue that whilst there is some evidence for emotional mimicry occurring in socially affiliative contexts [15], other evidence contradicts this and suggests that people also react with congruent emotional expressions in the absence of such social cues.

We hypothesise that the difference in findings across the studies investigating the relationship between facial mimicry and empathy can be explained by the following two factors:

1. Studies often not accounting for the differences in the empathetic ability across various demographic groups;
2. Studies following different designs [20] and, in particular, employing different instruments to quantify empathy.

While previous studies consistently report gender differences in empathy [21,22,23,24,25], results on age differences in empathy are mixed [26,27,28,29]. Furthermore, we found only one study considering ethnicity and suggesting there might be differences in the empathetic ability among people of different ethnic groups [25]. Hence, it is important to further verify if such differences exist, and thus, should be accounted for in future studies investigating the relationship between facial mimicry and empathy.

For measuring empathy, many studies (e.g., [30,31]) employed self-report measures such as the Empathy Scale [32], Questionnaire Measure of Emotional Empathy (QMEE) [33] and Reactivity Index [34]. According to Baron-Cohen and Wheelwright [35], these widely used self-report measures sometimes measure uncorrelated factors or processes broader than empathy. More recent studies (e.g., [28,36]) employed the performance-based Multifaceted Empathy Test [37], which measures both cognitive and affective empathy.

While there are differences in measuring empathy, facial mimicry has consistently been measured using electromyography (EMG) [19,30,31]. However, employing EMG for quantifying facial expressions is expensive and not practical in real-life settings as it requires electrodes to be attached to the participants’ faces. Hence, there is a need to investigate whether existing results still hold when measuring facial mimicry using alternative, more convenient methods.

This study, for the first time, uses a combination of Empathy Quotient (EQ) scores [35] to quantify empathy (considering both its emotional and cognitive aspects in a self-reported manner to be comparable with the majority of studies on empathy) and iMotion software [38] to quantify facial reactions from video recordings, rather than EMG, based on the AFFDEX algorithm developed by Affectiva [39] for testing the following hypotheses:

**Hypothesis** **1** (H1). *There is no significant difference in the empathetic ability (measured as EQ scores) of people across different demographic groups (based on gender, age and ethnicity).*

**Hypothesis** **2** (H2). *There is no correlation between measurable facial reactions to emotional stimuli (as detected by iMotions) and EQ scores.*

Two female and two male faces portraying the six basic emotions (fear, anger, joy, sad, disgust and surprise) and the neutral state were randomly selected from the Karolinska Directed Emotional Faces (KDEF) database and used as emotional stimuli in this study. The participants recruited for the study were exposed to these stimuli in an online study designed using iMotions software and presented to the participants through a web browser on their laptops. During the study, the participants faces were recorded using the participants’ laptop cameras and processed using the AFFDEX algorithm integrated into iMotions software to quantify facial expressions in response to the emotional stimuli.

**Figure 1:**
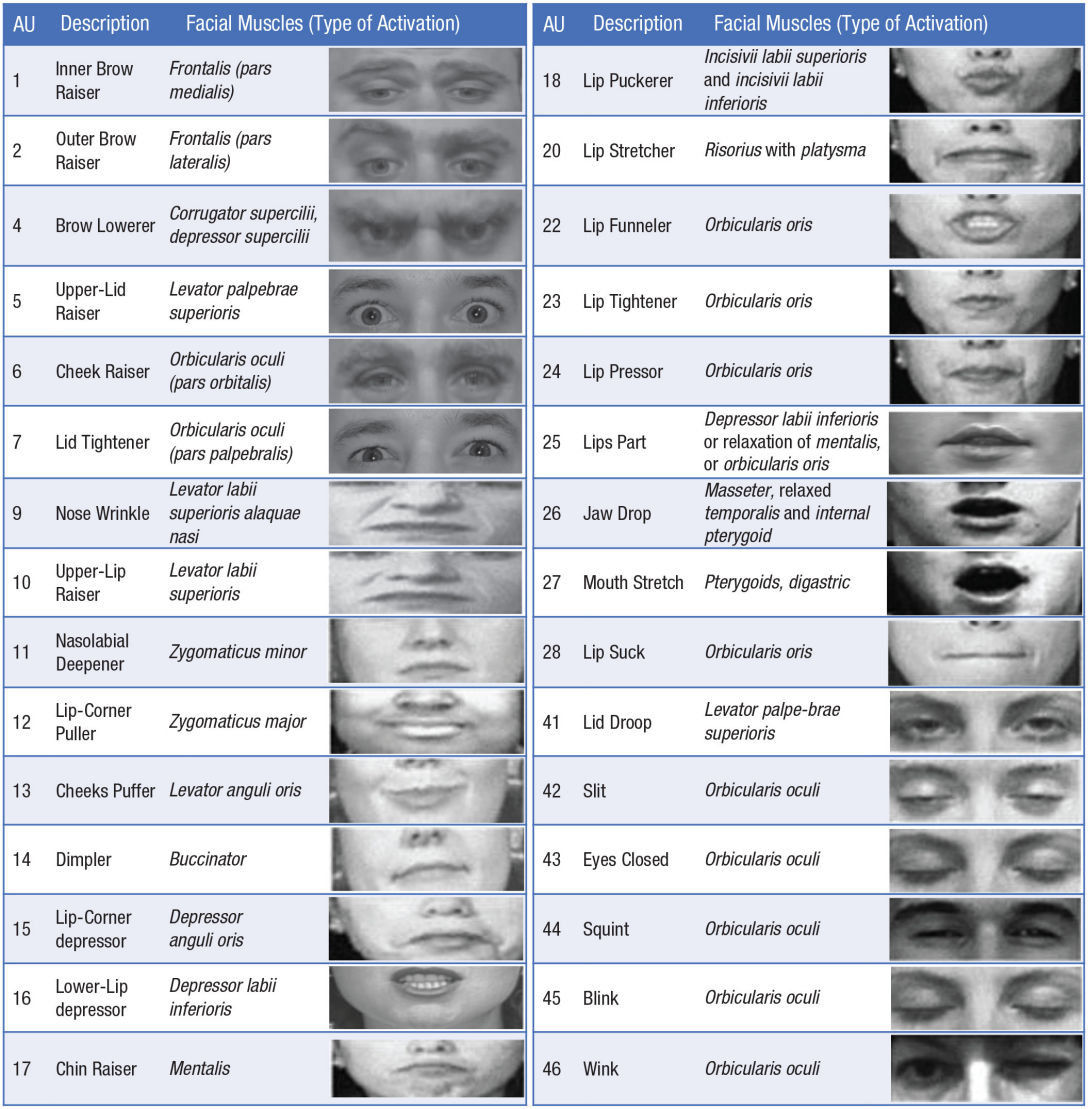
Facial Action Coding System [40] codes for adults. AU = action unit. Images for AUs are reproduced from [42].

The AFFDEX algorithm works in two stages: first, it detects facial expressions using deep learning based on the Facial Action Coding System (FACS) [40,41], and then maps the detected facial expressions to emotions as explained on the Affectiva web-pages (https://blog.affectiva.com/emotion-ai-101-all-about-emotion-detection-and-affectivas-emotion-metrics, accessed 21 April 2022). FACS represents a standardised classification system based on elementary components called action units (AUs). Each facial expression is a combination of AUs corresponding to the contraction of a distinct group of facial muscles. Figure 1 illustrates the facial AUs with corresponding facial movements.

It is important to note that the purpose of this study is to establish whether there is any facial response to any emotional stimulus that can be reliably correlated with EQ scores. The study does not attempt to accurately measure actual emotional states within other people (which indeed would be problematic as demonstrated in [43], for example). Instead of detecting what subjects feel when looking at various emotional stimuli, the study focuses on detecting facial movements to see if they mimic the ones present in emotional stimuli (i.e., detecting facial mimicry). Since the AFFDEX algorithm derives emotions based on detected facial expressions, in this study, we use the emotion labels as aggregates or semantic meaning of all the facial expressions detected in response to emotional stimuli. While we report results separately for facial landmark labels (constituting facial expressions) and derived emotion labels, we refer to both categories as “facial reactions” to quantify facial mimicry (not emotions).

The rest of the paper is organised as follows: Section 2 outlines the methodology of the presented study. The analysis results are reported and discussed in Section 3 and Section 4, respectively. Section 4 also discusses the suitability of various technologies for facial expression analysis in the context of holding online conversations. Section 5 concludes the paper.

## 2. Materials and Methods

### 2.1. Experimental Procedure

The study was approved by the Birmingham City University Research Ethics Committee. Eighty-seven participants (63 women, 24 men) took part in the study on a volunteer basis after providing their consent.

**Figure 2:**
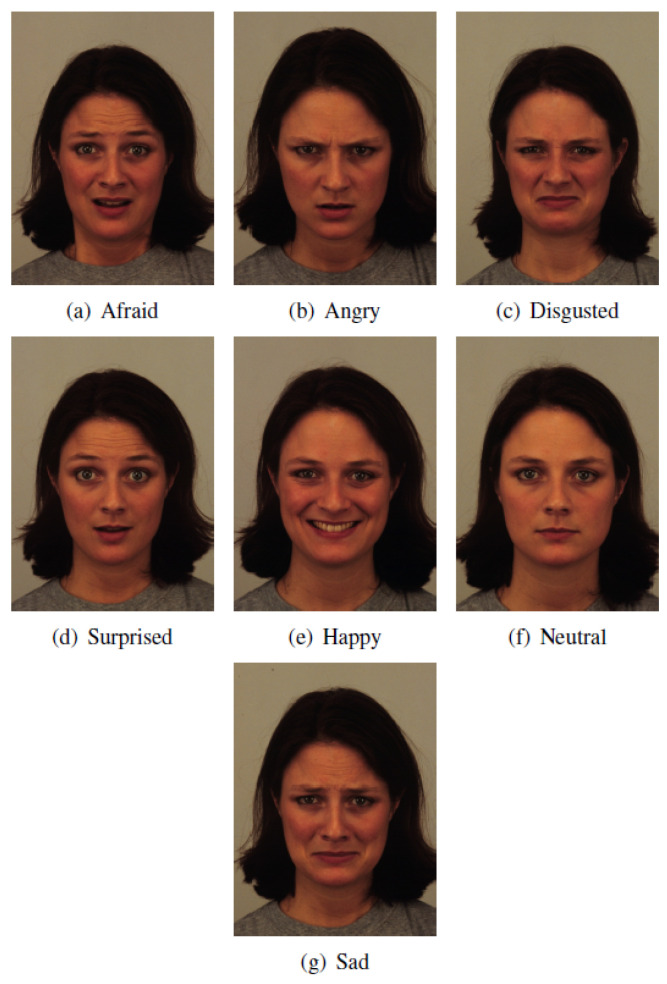
Karolinska Directed Emotional Faces database images example.

The study was implemented using iMotions software [44] and deployed on a cloud. The participants completed the study remotely using their web browser and laptop camera. In particular, the participants were asked to passively look at 28 images portraying six basic emotions (fear, anger, joy, sad, disgust, and surprise), along with a neutral face, of four individuals (two women, two men) randomly taken from the KDEF database. Figure 2 shows the facial expressions of one of the four individuals chosen for the study from the KDEF database.

**Figure 3:**
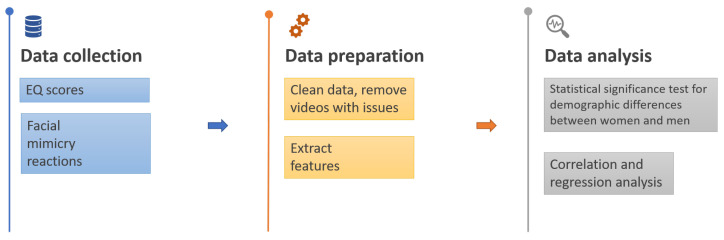
Architectural framework of the methodology.

The participants’ faces were recorded through their webcam during the exposure to the face images. To ensure that the participants were engaged with the images and in appropriate manner (e.g., not covering their face, talking, or eating at the same time), video captures were examined one by one and selected for the analysis using the quality metrics detailed in Section 2.3.2. The entire study procedure included the following stages: each participant was asked to fill in a short questionnaire about their demographic details, and then were shown a set of instructions and exposed to a sequence of facial images. The whole sequence was split into four blocks, each showing the six unique emotions in random order, followed by a neutral face. In each block, each facial image was shown for 10 s, followed by 8 s of a blank screen with a neutral background. After the first two blocks of faces and before the last two blocks of faces, each participant was asked to complete the EQ questionnaire comprising 40 empathy questions and 20 control questions, which can be found in Appendix A. The motivation behind putting the EQ questionnaire between showing the blocks of faces was to test whether reflecting on situations requiring empathy would induce facial reactions to emotional stimuli. Furthermore, this strategy allowed a break to prevent fatigue caused by participating in a long monotonic session. Overall, the study was taking about 20 min to complete, at the end of which, the data were automatically uploaded to a cloud. After the data were collected, they were preprocessed and analysed as described below in Section 2.3 and Section 2.4, respectively (Figure 3).

### 2.2. Theoretical Background

#### 2.2.1. Validation of iMotions

iMotions software performs automated facial expression analysis using computer vision algorithms. This non-intrusive approach allows for the collection of data in a wide variety of naturalistic environmental settings [44]. The automatic facial encoding varies depending on the facial expression engine, Affectiva AFFDEX (used in this study), Emotient FACET, or Noldus FaceReader, but they are all based on the same steps:

- *Face detection*: A classifier algorithm, e.g., Viola Jones Cascaded [45], identifies the position of the face in the video frame.
- *Feature detection*: A computer vision algorithm detects the landmarks of the face such as the corners of the eyebrow, corners of the mouth, and tip of the nose. In this step, an internal face model is created and it adapts to the movements of the face.
- *Feature classification*: Deep learning algorithms analyse the pixels in these regions and classify the facial expressions, which are then mapped to emotions.

The software returns numeric scores for facial expressions, AUs, and emotions, along with the value of the confidence level. The data returned can be visualised as raw or baseline-corrected. A baseline represents the neutral expression of the person, and allows for a relative analysis of the changes in the expression. For example, some people might have a baseline expression that is more stern. In such cases, without a baseline correction, the amount of frowning during the experiment could be under-/overestimated.

**Figure 4:**
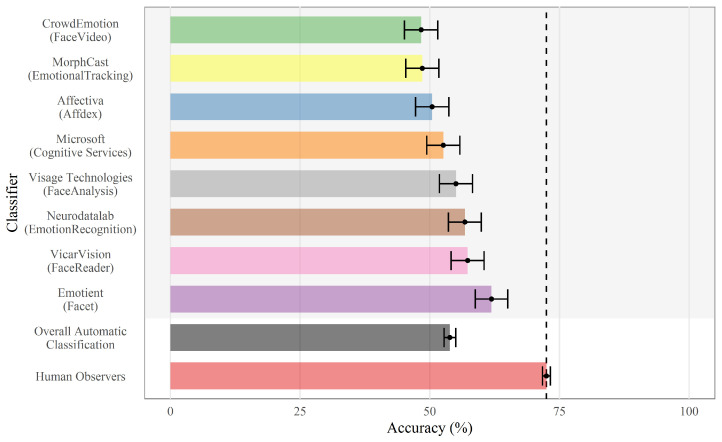
Mean true-positive emotion recognition performance of human observers and automatic classifiers. Reproduced from [48].

While there are other algorithms that have been demonstrated to be slightly more accurate than AFFDEX in detecting emotions [46,47], only iMotions software with the AFFDEX engine was available for this study, and [48] reports no statistically significant difference between AFFDEX and other automatic classifiers that were tested (Figure 4). Furthermore, this study is not concerned with accurate emotion detection but rather estimating the magnitude of facial reactions represented by facial landmarks to emotional stimuli. While there are no peer-reviewed studies reporting the accuracy of AFFDEX in detecting facial landmarks (and thus facial expressions), it is expected to perform on par with other deep-learning-based algorithms, which achieve accuracy scores of around 95% [49].

#### 2.2.2. Validation of Karolinska Directed Emotional Faces Database

The affective stimuli in the experiment are represented by the images of faces taken from the KDEF database [50]. This database contains a total of 4900 pictures of human facial expressions from 70 individuals and has been validated by Goeleven et al. [51]. It is important to mention that the experimental study of emotion requires stimuli evoking psychological and physiological reactions that vary consistently over the set of emotions. In the aforementioned validation study by Goeleven et al. [51], the images were evaluated on emotional content, intensity of the chosen emotion, and arousal with 272 participants. The authors of the validation study concluded that the KDEF database offers a valid set of input affective stimuli, with a mean biased hit rate of 72% (hit rate = index of the percentage of participants who correctly identified the KDEF target emotion).

While using staged expressions (such as the ones taken from the KDEF database) instead of spontaneous expressions can be seen as a limitation of this study (the hit rate for posed expressions might be inflated as they are easier to recognise), it provides a baseline collected in a controlled setting that can be used to compare against in future studies conducted in natural settings. Furthermore, using posed expressions can avert the ambiguity of spontaneous expressions, which is important for achieving the primary goal of this study, namely, evaluating the intensity of the reaction of the participants to different facial expressions in relation to their EQ scores.

Another limitation of using the KDEF database for this study is that it contains only white Caucasian adults. The database might fail to pick up cultural nuances; nonetheless, this is the only set of validated faces available to us and producing another set and validating it is a research study in its own right.

#### 2.2.3. Validation of Empathy Quotient Scale

The EQ score first proposed by Baron-Cohen and Wheelwright [35] is used in this study to measure the empathetic ability of the participants. The EQ score can be between 0 and 80, and it is calculated based on self-reported answers to 60 questions, 40 of which are designed to measure empathy, while the remaining 20 are control questions meant to distract the participant from persistent attention on empathy. To validate the proposed questionnaire, Baron-Cohen and Wheelwright [35] compared results to other related scales such as Friendship Questionnaire [52] and Autism Spectrum Quotient [53].

A self-reported method for the study of empathy includes inevitable limitations. For example, participants only assess the self-perceived individual beliefs about their empathy level, which can differ from the actual one. Nonetheless, the EQ is claimed to have enough external validity to be used in empathy-related studies. Furthermore, the purpose of this study is to see if facial mimicry can be a reliable predictor of EQ scores and thus be used as an objective measure of empathy in place of self-reported methods.

### 2.3. Data Description

#### 2.3.1. Data Collection

The participants accessed the study through a link to a website. After reading about the study and consenting to their data being collected, the participants were asked to provide their demographic details such as age band, gender, and ethnicity. Next, participants were provided with the instructions for the experiment and guided through several steps to allow video recording and screen capture. Finally, the participants were shown the first set of facial images, followed by the EQ questionnaire and second set of facial images. The participants’ facial activity was recorded during this final activity using their webcam, while their EQ score was captured through screen recording.

Once the video recordings were collected, they were processed offline into numerical values representing the probabilities of facial expressions and emotions identified by the AFFDEX algorithm embedded into iMotions software. The facial reaction data of each participant were then matched with the corresponding EQ score and demographic details.

#### 2.3.2. Data Preparation

The original dataset, overall containing more than 500k rows and 50 columns, had a total of 87 participants; however, only 81 participants had their EQ scores captured: 59 females, 22 males. These 81 participants were considered for the analysis of the differences in EQ scores across different demographic groups based on age, gender, and ethnicity. For the analysis of the relationship between EQ scores and facial mimicry, the number of included participants was reduced further as described below.

For the iMotion software to accurately classify facial expressions, the participant’s face and facial landmarks should be clearly visible. Therefore, a scale that indicates the confidence in facial data quality was defined as follows:

- 0—good quality;
- 1—minor issues such as subjects supporting their head with a hand, sometimes covering lips;
- 2—major issues such as subjects occasionally talking, eating, drinking or covering most of their face with hands;
- 3—no facial data available, e.g., subjects with camera off.

Based on this scale, the data were cleaned and filtered before the analysis. To achieve this, a new column was assigned to each participant with the quality value. When analysing the facial data, 50 participants with a good quality score of 0 were selected (36 females and 14 males).

### 2.4. Data Analysis

The EQ scores’ distribution and their differences across the demographic groups were analysed to test the first hypothesis. The facial reaction data distribution and correlation with the EQ scores were analysed to test the second hypothesis. Both analyses were performed in Python using appropriate libraries as described below.

#### 2.4.1. Difference in Empathy across Demographic Groups

The data were first visualised by plotting the distribution of EQ scores for males and females using the seaborn Python library. The independent *t* with Null hypothesis $\left(H_{0}\right)$: $mean_{1}=mean_{2}$ was then used to test for a significant difference between the mean of two unrelated groups (Male/Female, White ethnicity/Other, Age < *x*/Age > *x*). For this study, the researchpy [54] and scipy [55] Python libraries were used to perform the tests.

#### 2.4.2. Correlation between Facial Reactions and Empathy

iMotion software provides probability scores in the range 0–100 for 20 facial expressions (AUs) and seven core emotions (joy, anger, fear, disgust, contempt, sadness and surprise) for each frame in the recording of each participant. These values were analysed in relation to the stimuli (seven face expressions taken from the KDEF database) presented to the participants.

While 81 participants were included in the EQ scores analysis regardless of the quality score, only 50 participants with good quality scores were included in the facial data analysis. Both participants wearing glasses and those without glasses were included, and the difference across the two group was tested.

To establish whether there is an initial subconscious facial reaction to emotional stimuli, the maximum values were found for each emotion per each subject and stimulus. Then, all maximum values were averaged across subjects, and a set of bar charts was created (a plot per stimulus, i.e., KDEF face), with x-axis representing emotions (considered in this study as facial reactions or the semantic meaning of changes in facial expressions) and y-axis representing the average of the maximum scores. In addition, a set of boxplots was produced for the same to demonstrate the spread of the maximum scores across all the subjects.

To test whether facial reactions can be predictors of empathy (which is important to know in the context of developing empathy training tools, for example), three sets of three regression models were fitted to predict the EQ score using machine learning algorithms. The first set of models was built using the scores provided for both emotion and facial landmark labels, the second — for emotion labels only, and the third — for facial landmark labels only. While the inclusion of both emotion and facial landmark scores to predict the EQ score is expected to improve the predictive ability of the models, their separation allows us to check whether predicting the EQ score based on emotion labels only provides results similar to those when only facial landmark labels are used. The similarity in results would justify the use of emotion labels in place of facial landmark labels to represent facial reactions when looking at the relationship between empathy and facial mimicry.

In all three models in each of the three sets, the EQ score column was taken as the dependent variable (*y*), while the multiple input features (i.e., emotion and/or landmark scores) were taken as independent variables ($x_{i}$). The first two models were fitted using the LinearRegression function from the sklearn Python library [56]. The predictions of the first model were made over the full dataset, whereas the second model was trained using a training set (70% of all data selected randomly), and predictions were made over a test set (i.e., using the remaining 30% unseen data). The third model was fitted using the ElasticNet function from the sklearn library, which extends the LinearRegression function by combining the L1 and L2 penalty functions during the training process. In this case, the data were scaled using the StandardScaler function from the sklearn library; k-fold cross-validation and grid search were employed for hyperparameter tuning.

After fitting the three models in each of the three sets, their performance was analysed and a further statistical investigation was carried out using the statsmodels Python library [57]. Correlation coefficients between the EQ and facial reaction scores were also calculated.

To establish whether it is possible to predict low- and high-emphatic individuals based on facial reactions, the participants were divided into two groups using the median EQ score (45) as a cut-off point. For mimicry reactions, the analysis focused on joy/anger emotional reactions to happy/angry stimuli and the maximum reactions (in the first two seconds from the onset of the stimuli) of the two groups were compared using the Mann–Whitney U Test [58]. For facial reaction in general (all emotions), the two groups were compared considering the average across the maximum values of each emotion.

To check whether there was a significant difference in the detected reaction values between the participants wearing glasses and not, a *t*-test was performed on the average of all maximum reaction values across all emotions.

## 3. Results

### 3.1. Difference in Empathy across Demographic Groups

Eighty-one participants completed the questionnaires in full; 59 identified their gender as female, 22 as male. The mean EQ score is 47 (SD = 10). The main ethnicity is *White British* (67.9%), followed by *White Other* (13.6%) and *Black African* (8.6%).

**Figure 5:**
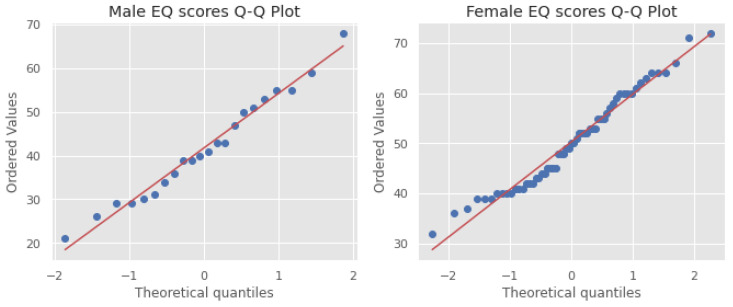
Q–Q plots of empathy quotient (EQ) scores for males and females.

**Figure 6:**
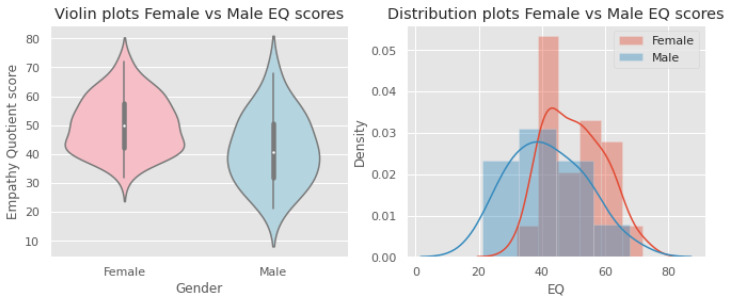
Violin plots (**left**) and distributions (**right**) of EQ scores for males and females.

The EQ score distributions for males and females are Gaussian, which is confirmed by the Q–Q plots and Shapiro–Wilk test scores (males: statistics = 0.979, *p* = 0.900; females: statistics = 0.969, *p* = 0.133). Figure 5 and Figure 6 show the Q–Q plots and distributions, respectively.

Four *t*-tests were performed to test the null hypothesis that the two demographic groups (Male vs. Females, White British vs. Other, All white vs. Other, Aged 18–34 vs. Other) have the same EQ mean value and there is no statistically significant differences between them. The null hypothesis of identical average scores cannot be rejected if the *p*-value is higher than a threshold, which is set to 1% in this study. If the *p*-value is smaller than the threshold, then the null hypothesis of equal averages can be rejected.

The four demographic groups were defined as follows: In the first *t* (Male vs. Females), there were 22 participants in the male group (Mean EQ score = 41.77, SD = 11.9) and 59 participants in the female group (Mean = 50.28, SD = 9.39). In the second *t* (White British vs. Other), there were 55 White British individuals (Mean = 49.09, SD = 11.36) and 26 individuals of other ethnicity (including Pakistani, Indian, Black Caribbean, Mixed White/Black Caribbean, Bangladeshi, White Irish, Arab, Black African, and White other) (Mean = 45.61, SD = 9.19). The third *t* (White ethnicity vs. Others) included 56 participants in the first (White) group (Mean = 48.75, SD = 11.54) and 25 participants in the second (Other) group (Mean = 46.24, SD = 8.80). The final *t*-test (Aged 18–34 vs. Other) had 21 individuals aged 18–34 (Mea n = 44.47, SD = 11.73) and 60 participants aged 35+ (Mean = 49.20, SD = 10.24).

The following results were obtained: The *t* for gender (Male vs. Female) indicated a significant difference (*p*-value = 0.001 < 1%, *t*-value = − 3.35, Cohen’s d = −0.83, Pearson’s r = 0.35). There was no statistical significance in the difference between White British vs. Other (*p*-value = 0.177 > 1%, *t*-value = 1.36, Cohen’s d = 0.32, Pearson’s r = 0.15) or White vs. Other (*p*-value = 0.336 > 1%, *t*-value = 0.96, Cohen’s d = 0.23, Pearson’s r = 0.10). The difference between 18–34 years old participants vs. Others was also not significant (*p*-value = 0.083 > 1%, *t*-value = −1.75, Cohen’s d = −0.44, Pearson’s r = 0.19).

The results confirm the finding by previous studies that women report higher EQ scores than men. The average EQ score for women is 50 (SD = 9.3), while the average EQ score for men is 41 (SD = 11.9). At the same time, the EQ scores were not significantly different among people of different ethnicity or age, as well as before and after completing the EQ questionnaire.

### 3.2. Correlation between Facial Reactions and Empathy

**Figure 7:**
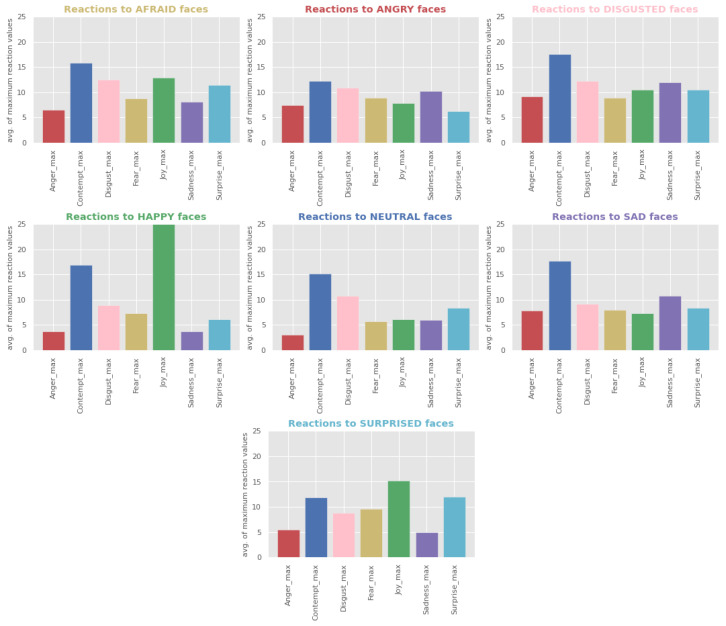
Facial reactions vs. stimuli bar plots generated by taking the average of the maximum values for each reaction and stimulus across all subjects. Each plot represents the data for one type of emotional stimuli, with x-axis representing the types of reaction and y-axis representing the average of the maximum reaction probability scores.

**Figure 8:**
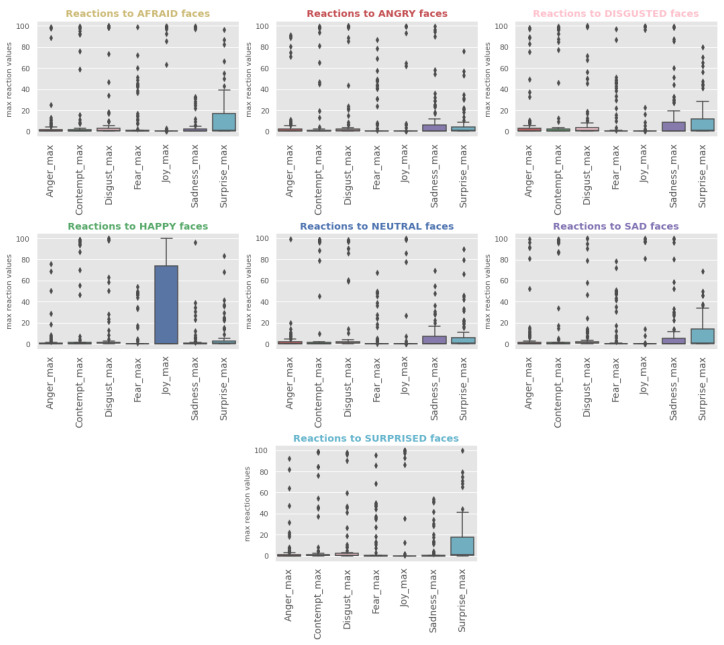
Facial reactions vs. stimuli boxplots generated by considering all the maximum values for each reaction and stimulus. Each plot represents the data for one type of emotional stimuli, with x-axis representing the types of reaction and y-axis representing the maximum reaction probability scores.

As a first insight into the facial data, it is useful to establish which stimuli (KDEF images) evoke stronger reactions, whether the same stimuli evoke respective emotions, how reactions across different stimuli relate to the reaction to neutral face, and whether there is a dominant reaction. One can notice from Figure 7 a high average value for the contempt reaction across all stimuli and that the most intense reaction is joy generated when looking at the happy stimulus. The latter is also clearly visible in Figure 8, showing the spread of the max reaction values across all the subjects per each stimuli. At the same time, Figure 8 demonstrates that the high average value for contempt is explained by only a few subjects, while facial expressions making up surprise are mildly activated across all the stimuli except the happy stimulus, with the highest intensity demonstrated when exposed to surprised faces.

**Figure 9:**
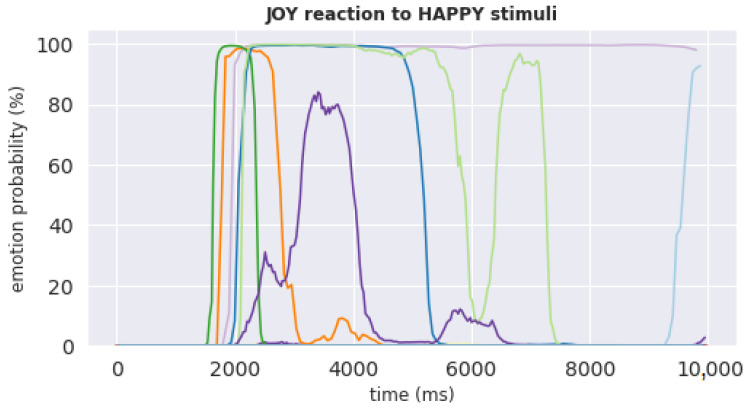
Joy reaction of all 50 subjects during the 10 s from the onset of being shown the happy stimulus. X-axis represents time (ms) and y-axis represents joy probability scores (%). Each uniquely colored line represents reaction of one subject, with the majority of subjects having near-zero reaction.

Figure 9 shows the time-series of the joy reaction of all 50 subjects during the 10 s from the onset of being shown the happy stimulus. The joy reaction to happy stimulus was selected to check whether our results are consistent with those reported by Dimberg et al. [10], namely, that “pictures of happy faces spontaneously evoke increased zygomatic major muscle activity ... after only 500 ms of exposure”. It can be noticed from Figure 9 that while many subjects who had a strong reaction to happy stimulus indeed had a spike in reaction at the start of the exposure period, these spikes tend to happen after nearly 2000 ms of exposure. This delay compared to the finding by Dimberg et al. [10] may be explained by the delay of iMotions software in registering facial reactions captured by participants’ video cameras over the Internet.

Next, we analysed the relationship between facial reactions and empathy. It can be noticed from Table 1 that there is no strong correlation (i.e., no values > 0.5) between facial reactions and the EQ score. To further analyse this relationship, three sets of multiple linear regression models were fitted as described in Section 3.2.

To have a full description of regression coefficients, the statsmodels library [57] was used as it provides more details on the statistical significance of the coefficients than the sklearn library [56]. Table 2 lists the regression results for the model trained using all emotional and facial landmark scores. It can be noticed from the table that while the emotions do not have significant *p*-values, some facial landmarks do (“Brow Furrow”, “Inner Brow Raise”, “Jaw Drop”, “Lip Press”, “Lid Tighten”, and “Nose Wrinkle”).

Table 3 lists the R-squared and mean squared error (MAE) values for all the three regression models in each of the three sets built as described in Section 3.2. It can be noticed from the table that while none of the models can accurately predict EQ scores (as indicated by low R-squared values), the models trained on both emotion and facial landmark scores outperform the models trained on either emotion scores or facial landmark scores, with the models trained on only emotion scores performing the worst. The much lower R-squared values achieved by the models trained using only emotion scores compared to those achieved by the models trained using only facial landmark scores (3% vs. 16% for tuned models) suggest that the mapping from facial expressions to emotions provided by existing facial recognition software is not reliable and should not be used in the context of detecting or training one’s empathetic ability. Overall, the results confirm a weak correlation between facial reactions and EQ scores, and indicate that EQ scores cannot be predicted with high accuracy using facial reactions to emotional stimuli.

Finally, to allow the comparison of our results to those reported in other studies based on the categorical split of people into low- and high-empathetic individuals, the median EQ score (45) was used to separate the participants into two groups: 26 high-empathetic and 24 low-empathetic individuals. When analysing the joy/anger reactions to happy/angry stimuli, we noticed that the values were not distributed normally; hence, the Mann–Whitney U Test [58] was employed to test the statistical difference between the groups. The maximum values of joy and anger in the first two seconds from when the respective stimulus appeared were taken into consideration. The results of the test were not significant (joy *p*-value = 0.21, anger *p*-value = 0.15). When repeating the same experiment with the average value of all the facial reaction scores, the result was again non-significant with a *p*-value of 0.81.

No significant difference was found in facial reactions between participants wearing and not wearing glasses (*p*-value = 0.22).

## 4. Discussion

The average EQ score is 47 (SD = 10), which is comparable to the scores reported for other Western countries [59]. According to the results of this study, females score higher than males in the EQ questionnaire. This result confirms what has been found in previous studies [21,22,23,24,25]. At the same time, in contrast to previous studies reporting age differences in empathy [25,26,27,28,29], we found no difference in the empathetic ability among people of different ages; however, this might be due to not considering age groups at a more granular level as other studies did. Similarly, our finding that there is no difference in the empathetic ability among people of different ethnic groups contradicts that of Sommerlad et al. [25], who found greater empathetic concern associated with non-white ethnicity. Nonetheless, empathy is a process that happens mainly as an internal reaction, and it might be hard to determine the validity of the measurements when these are self-assessed, as in the case of the EQ questionnaire [21].

When analysing the facial data results, we noticed relatively strong contempt and/or surprise expressions across many subjects even for neutral stimuli, perhaps reflecting the need for concentration to perform the study and/or reaction of facing unknown. Regarding mimicry, joy as a reaction to happy stimuli appears to be the only strong reaction with high reaction values in many subjects.

Our results of the correlation analysis between facial reactions to stimuli (KDEF images) and EQ scores indicate that there is no strong correlation between facial reactions and empathy or difference in reactions between high- and low-empathetic individuals. These results contradict those reported in previous studies such as [13,30,31,60]. This disagreement can be due to the inability of iMotions software, which uses deep learning facial recognition methods, to detect expressions that are weak and hardly discernible by observation. When facial muscles are activated, they usually create the movement of facial landmarks that iMotions can detect, but when this muscle activation is very weak or transient, the movement may not appear on the face as an expression. iMotions provide the probability values representing the likelihood of each emotion being expressed based on the movement of facial landmarks. Out of the 50 participants that were selected as having good quality recordings, only three had a reaction value higher than 50% (two of them not wearing glasses). This means that the majority of the participants did not show a consistent and significant reaction.

While the EMG approach adopted by other studies can be more sensitive to minor changes in facial expressions, it is not practical in real-world scenarios. At the same time, albeit easy and cheap to deploy, the current facial recognition technology based on the facial action coding scheme may be unsuitable for capturing mimicry as an objective measure of empathy [61]. This motivates the need for improving the emotion detection ability of deep learning methods applied to video streams.

Another interpretation of our results can be that facial expressions shown through a video camera may not be perceived by the receiver as opposed to face-to-face communication. Further experiments are required to understand the limiting factors of capturing and perceiving facial mimicry in virtual environments. For example, it would be interesting to compare the results of translating facial expressions observed through a video camera into emotions performed by facial recognition software and human participants.

Finally, the observed weak reactions of the participants in our study could be the artificial setting of the experiment: the faces shown to the participants were static pictures of expressions posed by various people and not natural expressions in a context. A more realistic study could include dynamic stimuli or recording participants’ faces during a conversation, where a participant and an experimenter discuss stories evoking different emotions; then, the faces of the two speakers could be overlaid to check if there are any matches in facial expressions. It would be interesting to compare the results between the controlled experiment reported in this paper and that performed in a more natural setting. As a compromise between the two extreme scenarios, dynamic stimuli such as videos can be employed in a controlled way, given that some studies have found a stronger emotion-specific response to these stimuli as opposed to static stimuli such as images [62,63].

One of the limitations of this study is a larger number of females compared to males and an overall small cohort of participants; the required number for statistical power as defined by Cohen [64] would be 128. Further limitations of the study include not having control over subject sessions. The experiments were performed online given the COVID-19 pandemic. At the same time, the collected data represent a more realistic scenario of the post-pandemic world and thus highlight the issues that one should expect when applying facial recognition technologies in real settings. Similarly, while including subjects wearing glasses may have contributed to obtaining weaker signals, the obtained results are representative of real-life settings. In fact, all such variations in experimental setup can be summarised as “not-fully understood factors” noted by Holland et al. [65] that contribute to the ongoing debate on the linkage between facial mimicry, empathetic ability, and emotion recognition.

## 5. Conclusions

The relationship between facial mimicry/expressions and empathetic ability has been long discussed in scientific literature. As mimicry appears to be a subtle and automatic response during interpersonal interactions, it has to be detected with very sensitive measurements such as EMG, which is not practical in many real-life scenarios. At the same time, the right method to measure empathy (at the physiological and psychological levels) is also debated. Therefore, studying the relationship between empathy and facial mimicry is a challenging task, and results appear to depend on the study design and instruments employed to measure both empathy and facial reactions to stimuli. The aim of this exploratory study was to apply machine learning algorithms and statistical methods to analyse this relationship in the online setting. The proposed method can be interpreted as a new approach to the problem, using novel tools such as facial recognition technology (iMotions software in this case) that allows facial mimicry and emotion analysis during real-life online (virtual) interpersonal communication. While some results of this study confirm those reported in previous studies (such as the difference in EQ scores between females and males), other claims could not be verified (such as the correlation between empathy and facial reactions).

The limitations of this work have left several opportunities for future research. On the one hand, repeating the experiment in a laboratory under experimenter supervision rather than letting subjects participate online from their homes may help increase data quality. On the other hand, collecting data during real-life interpersonal interactions (e.g., during online mental health therapy sessions) may provide more realistic results and reveal the limitations of the latest facial recognition technology.

## Figures and Tables

**Table 1:** Correlation coefficients between EQ score and facial reactions (sorted in descending order).

Facial Reaction	Correlation Coefficients
Lip Press_max	0.284270
Dimpler_max	0.231334
Smirk_max	0.226920
Contempt_max	0.199976
Lip Stretch_max	0.192604
Smile_max	0.161596
Joy_max	0.155527
Anger_max	0.127649
Lip Pucker_max	0.119811
Lip Suck_max	0.112801
Eye Closure_max	0.112600
Cheek Raise_max	0.105514
Chin Raise_max	0.072400
Eye Widen_max	0.070983
Sadness_max	0.067079
Fear_max	0.035937
Brow Raise_max	0.031838
Surprise_max	0.021879
Mouth Open_max	0.014010
Brow Furrow_max	−0.012756
Lip Corner Depressor_max	−0.071880
Jaw Drop_max	−0.138609
Lid Tighten_max	−0.180354
Inner Brow Raise_max	−0.196593
Upper Lip Raise_max	−0.200510
Disgust_max	−0.223726
Nose Wrinkle_max	−0.266633

**Table 2:** Ordinary least squares regression results.

	Coef	Std Err	t	P >|t|	[0.025	0.975]
const	48.5614	0.760	63.922	0.000	47.069	50.053
Anger	0.4218	0.625	0.675	0.500	−0.806	1.649
Contempt	−2.6834	1.096	−2.448	0.015	−4.836	−0.531
Disgust	0.7851	0.413	1.902	0.058	−0.026	1.596
Fear	−0.1177	0.194	−0.606	0.545	−0.499	0.264
Joy	2.6164	9.383	0.279	0.780	−15.812	21.045
Sadness	−0.2851	0.266	−1.072	0.284	−0.807	0.237
Surprise	−0.2870	0.404	−0.710	0.478	−1.081	0.507
Brow Furrow	0.1652	0.055	2.977	0.003	0.056	0.274
Brow Raise	0.0568	0.086	0.658	0.511	−0.113	0.226
Cheek Raise	−0.5322	1.002	−0.531	0.596	−2.501	1.436
Chin Raise	−0.2896	0.864	−0.335	0.738	−1.987	1.408
Dimpler	16.0359	20.694	0.775	0.439	−24.607	56.679
Eye Closure	0.0736	0.033	2.213	0.027	0.008	0.139
Eye Widen	0.0040	0.053	0.076	0.940	−0.101	0.109
Inner Brow Raise	−0.5522	0.184	−3.005	0.003	−0.913	−0.191
Jaw Drop	−14.2914	2.925	−4.886	0.000	−20.035	−8.547
Lip Corner Depressor	−252.6221	127.722	−1.978	0.048	−503.471	−1.774
Lip Press	59.9338	18.210	3.291	0.001	24.169	95.699
Lip Pucker	5.6872	15.657	0.363	0.717	−25.064	36.438
Lip Stretch	−53.8102	100.975	−0.533	0.594	−252.126	144.506
Lip Suck	−0.0005	0.101	−0.005	0.996	−0.198	0.197
Lid Tighten	−1.4160	0.298	−4.756	0.000	−2.001	−0.831
Mouth Open	0.1907	0.100	1.909	0.057	−0.006	0.387
Nose Wrinkle	−2.7569	0.933	−2.954	0.003	−4.590	−0.924
Smile	0.2902	0.642	0.452	0.651	−0.971	1.552
Smirk	2.3048	0.898	2.568	0.010	0.542	4.068
Upper Lip Raise	0.4662	0.282	1.651	0.099	−0.088	1.021

**Table 3:** Performance of the predictive models.

Model	R-Squared	MAE
Linear regression using all data (emotions and facial landmarks)	0.26	8.13
Linear regression train–test split (emotions and facial landmarks)	0.19	7.86
Tuned linear regression (emotions and facial landmarks)	0.24	8.74
Linear regression using all data (emotions)	0.03	9.80
Linear regression train–test split (emotions)	0.03	8.99
Tuned linear regression (emotions)	0.03	9.90
Linear regression using all data (facial landmarks)	0.16	8.85
Linear regression train–test split (facial landmarks)	0.09	8.35
Tuned linear regression (facial landmarks)	0.16	9.18

## Data Availability

Readers are advised to contact the corresponding author to discuss data access.

## Abbreviations

The following abbreviations are used in this manuscript:

EQ	Empathy Quotient
EMG	Electromyography

## Appendix A. Empathy Quotient Questionnaire

1. I can easily tell if someone else wants to enter a conversation.
2. I prefer animals to humans.
3. I try to keep up with the current trends and fashions.
4. I find it difficult to explain to others things that I understand easily, when they don’t understand it the first time.
5. I dream most nights.
6. I really enjoy caring for other people.
7. I try to solve my own problems rather than discussing them with others.
8. I find it hard to know what to do in a social situation.
9. I am at my best first thing in the morning.
10. People often tell me that I went too far in driving my point home in a discussion.
11. It doesn’t bother me too much if I am late meeting a friend.
12. Friendships and relationships are just too difficult, so I tend not to bother with them.
13. I would never break a law, no matter how minor.
14. I often find it difficult to judge if something is rude or polite.
15. In a conversation, I tend to focus on my own thoughts rather than on what my listener might be thinking.
16. I prefer practical jokes to verbal humor.
17. I live life for today rather than the future.
18. When I was a child, I enjoyed cutting up worms to see what would happen.
19. I can pick up quickly if someone says one thing but means another.
20. I tend to have very strong opinions about morality.
21. It is hard for me to see why some things upset people so much.
22. I find it easy to put myself in somebody else’s shoes.
23. I think that good manners are the most important thing a parent can teach their child.
24. I like to do things on the spur of the moment.
25. I am good at predicting how someone will feel.
26. I am quick to spot when someone in a group is feeling awkward or uncomfortable.
27. If I say something that someone else is offended by, I think that that’s their problem, not mine.
28. If anyone asked me if I liked their haircut, I would reply truthfully, even if I didn’t like it.
29. I can’t always see why someone should have felt offended by a remark.
30. People often tell me that I am very unpredictable.
31. I enjoy being the center of attention at any social gathering.
32. Seeing people cry doesn’t really upset me.
33. I enjoy having discussions about politics.
34. I am very blunt, which some people take to be rudeness, even though this is unintentional.
35. I don’t find social situations confusing.
36. Other people tell me I am good at understanding how they are feeling and what they are thinking.
37. When I talk to people, I tend to talk about their experiences rather than my own.
38. It upsets me to see an animal in pain.
39. I am able to make decisions without being influenced by people’s feelings.
40. I can’t relax until I have done everything I had planned to do that day.
41. I can easily tell if someone else is interested or bored with what I am saying.
42. I get upset if I see people suffering on news programs.
43. Friends usually talk to me about their problems as they say that I am very understanding.
44. I can sense if I am intruding, even if the other person doesn’t tell me.
45. I often start new hobbies, but quickly become bored with them and move on to something else.
46. People sometimes tell me that I have gone too far with teasing.
47. I would be too nervous to go on a big rollercoaster.
48. Other people often say that I am insensitive, though I don’t always see why.
49. If I see a stranger in a group, I think that it is up to them to make an effort to join in.
50. I usually stay emotionally detached when watching a film.
51. I like to be very organized in day-to-day life and often makes lists of the chores I have to do.
52. I can tune into how someone else feels rapidly and intuitively.
53. I don’t like to take risks.
54. I can easily work out what another person might want to talk about.
55. I can tell if someone is masking their true emotion.
56. Before making a decision, I always weigh up the pros and cons.
57. I don’t consciously work out the rules of social situations.
58. I am good at predicting what someone will do.
59. I tend to get emotionally involved with a friend’s problems.
60. I can usually appreciate the other person’s viewpoint, even if I don’t agree with it.

## References

[1] Woolley A.W., Chabris C.F., Pentland A., Hashmi N., Malone T.W. (2010). Evidence for a Collective Intelligence Factor in the Performance of Human Groups. Science.

[2] Salomon G. (1997). Distributed Cognitions: Psychological and Educational Considerations.

[3] Howick J., Moscrop A., Mebius A., Fanshawe T.R., Lewith G., Bishop F.L., Mistiaen P., Roberts N.W., Dieninytė E., Hu X.Y. (2018). Effects of empathic and positive communication in healthcare consultations: A systematic review and meta-analysis. J. R. Soc. Med..

[4] Szilágyi A.K., Dioszeghy C., Benczúr L., Varga K. (2007). Effectiveness of Psychological Support based on Positive Suggestion with the Ventilated Patient. Eur. J. Ment. Health.

[5] Rakel D.P., Hoeft T.J., Barrett B.P., Chewning B.A., Craig B.M., Niu M. (2009). Practitioner Empathy and the Duration of the Common Cold. Fam. Med..

[6] Menendez M.E., Chen N.C., Mudgal C.S., Jupiter J.B., Ring D. (2015). Physician Empathy as a Driver of Hand Surgery Patient Satisfaction. J. Hand Surg..

[7] Cahill J., Paley G., Hardy G. (2013). What do patients find helpful in psychotherapy? Implications for the therapeutic relationship in mental health nursing. J. Psychiatr. Ment. Health Nurs..

[8] Sweeney A., Clement S., Filson B., Kennedy A. (2016). Trauma-informed mental healthcare in the UK: What is it and how can we further its development?. Ment. Health Rev. J..

[9] Greenberg D.M., Warrier V., Allison C., Baron-Cohen S. (2018). Testing the Empathizing–Systemizing theory of sex differences and the Extreme Male Brain theory of autism in half a million people. Proc. Natl. Acad. Sci. USA.

[10] Dimberg U., Thunberg M., Elmehed K. (2000). Unconscious Facial Reactions to Emotional Facial Expressions. Psychol. Sci..

[11] Watson J.C., Greenberg L.S. (2009). Empathic Resonance: A Neuroscience Perspective. The Social Neuroscience of Empathy.

[12] Borgomaneri S., Bolloni C., Sessa P., Avenanti A. (2020). Blocking facial mimicry affects recognition of facial and body expressions. PLoS ONE.

[13] Chartrand T.L., Bargh J.A. (1999). The chameleon effect: The perception–behavior link and social interaction.-PsycNET. J. Personal. Soc. Psychol..

[14] Hatfield E., Carpenter M., Rapson R.L. (2014). Emotional contagion as a precursor to collective emotions. Collect. Emot. Perspect. Psychol. Philos. Sociol..

[15] Hess U., Fischer A.H. (2016). Emotional Mimicry in Social Context.

[16] Stel M. (2016). The role of mimicry in understanding the emotions of others. Emotional Mimicry in Social Context.

[17] Wróbel M., Piórkowska M., Rzeczkowska M., Troszczyńska A., Tolopilo A., Olszanowski M. (2021). The ‘Big Two’ and socially induced emotions: Agency and communion jointly influence emotional contagion and emotional mimicry. Motiv. Emot..

[18] Peng S., Zhang L., Hu P. (2021). Relating self-other overlap to ingroup bias in emotional mimicry. Soc. Neurosci..

[19] Wingenbach T.S.H., Brosnan M., Pfaltz M.C., Peyk P., Ashwin C. (2020). Perception of Discrete Emotions in Others: Evidence for Distinct Facial Mimicry Patterns. Sci. Rep..

[20] Wróbel M., Imbir K.K. (2019). Broadening the Perspective on Emotional Contagion and Emotional Mimicry: The Correction Hypothesis. Perspect. Psychol. Sci..

[21] Eisenberg N., Lennon R. (1983). Sex Differences in Empathy and Related Capacities. Psychol. Bull..

[22] Hoffman M.L. (1977). Sex differences in empathy and related behaviors. Psychol. Bull..

[23] Davis M.H. (1983). Measuring individual differences in empathy: Evidence for a multidimensional approach. J. Pers. Soc. Psychol..

[24] Christov-Moore L., Simpson E.A., Coudé G., Grigaityte K., Iacoboni M., Ferrari P.F. (2014). Empathy: Gender effects in brain and behavior. Neurosci. Biobehav. Rev..

[25] Sommerlad A., Huntley J., Livingston G., Rankin K.P., Fancourt D. (2021). Empathy and its associations with age and sociodemographic characteristics in a large UK population sample. PLoS ONE.

[26] Sun B., Luo Z., Zhang W., Li W., Li X. (2018). Age-related differences in affective and cognitive empathy: Self-report and performance-based evidence. Aging Neuropsychol. Cogn..

[27] Beadle J.N., de la Vega C.E. (2019). Impact of Aging on Empathy: Review of Psychological and Neural Mechanisms. Front. Psychiatry.

[28] Ziaei M., Oestreich L., Reutens D.C., Ebner N.C. (2021). Age-related differences in negative cognitive empathy but similarities in positive affective empathy. Brain Struct. Funct..

[29] Pollerhoff L., Stietz J., Depow G.J., Inzlicht M., Kanske P., Li S.C., Reiter A.M.F. (2022). Investigating adult age differences in real-life empathy, prosociality, and well-being using experience sampling. Sci. Rep..

[30] Sonnby-Borgström M., Jönsson P., Svensson O. (2003). Emotional Empathy as Related to Mimicry Reactions at Different Levels of Information Processing. J. Nonverbal. Behav..

[31] Rymarczyk K., Żurawski Ł., Jankowiak-Siuda K., Szatkowska I. (2016). Emotional Empathy and Facial Mimicry for Static and Dynamic Facial Expressions of Fear and Disgust. Front. Psychol..

[32] Hogan R. (1969). Development of an empathy scale. J. Consult. Clin. Psychol..

[33] Mehrabian A., Epstein N. (1972). A measure of emotional empathy1. J. Pers..

[34] Davis M.H. (1980). A Multidimensional Approach to Individual Differences in Empathy. JSAS Cat. Sel. Doc. Psychol..

[35] Baron-Cohen S., Wheelwright S. (2004). The Empathy Quotient: An Investigation of Adults with Asperger Syndrome or High Functioning Autism, and Normal Sex Differences. J. Autism Dev. Disord..

[36] Quinde-Zlibut J.M., Williams Z.J., Gerdes M., Mash L.E., Heflin B.H., Cascio C. (2021). Multifaceted empathy differences in children and adults with autism. Sci. Rep..

[37] Dziobek I., Rogers K., Fleck S., Bahnemann M., Heekeren H.R., Wolf O.T., Convit A. (2008). Dissociation of cognitive and emotional empathy in adults with Asperger syndrome using in the Multifaceted Empathy Test. J. Autism. Dev. Disord..

[38] iMotions (2017). Facial Expression Analysis—iMotions Software Solution. https://imotions.com/biosensor/fea-facial-expression-analysis/.

[39] Affectiva (2020). Solutions-Affectiva. https://www.affectiva.com/what/products.

[40] Ekman P., Friesen W. (1978). Manual for the Facial Action Coding System.

[41] Ekman P., Friesen W.V., Hager J.C. (2002). Facial Action Coding System: The Manual.

[42] Kanade T., Cohn J.F., Tian Y. Comprehensive database for facial expression analysis. Proceedings of the Proceedings Fourth IEEE International Conference on Automatic Face and Gesture Recognition (Cat. No. PR00580).

[43] Barrett L.F., Adolphs R., Marsella S., Martinez A.M., Pollak S.D. (2019). Emotional Expressions Reconsidered: Challenges to Inferring Emotion From Human Facial Movements. Psychol. Sci. Public Interest.

[44] iMotions (2020). Facial Expression Analysis Guide. https://imotions.com/guides/.

[45] Viola P., Jones M. Rapid object detection using a boosted cascade of simple features. Proceedings of the 2001 IEEE Computer Society Conference on Computer Vision and Pattern Recognition, CVPR 2001.

[46] Stöckli S., Schulte-Mecklenbeck M., Borer S., Samson A.C. (2018). Facial expression analysis with AFFDEX and FACET: A validation study. Behav. Res. Methods.

[47] Kulke L., Feyerabend D., Schacht A. (2020). A Comparison of the Affectiva iMotions Facial Expression Analysis Software with EMG for Identifying Facial Expressions of Emotion. Front. Psychol..

[48] Dupré D., Krumhuber E.G., Küster D., McKeown G.J. (2020). A performance comparison of eight commercially available automatic classifiers for facial affect recognition. PLoS ONE.

[49] Zhang K., Zhang Z., Li Z., Qiao Y. (2016). Joint Face Detection and Alignment Using Multitask Cascaded Convolutional Networks. IEEE Signal Process. Lett..

[50] Lundqvist D., Flykt A., Öhman A. (1998). The Karolinska Directed Emotional Faces-KDEF, CD ROM from Department of Clinical Neuroscience, Psychology Section.

[51] Goeleven E., De Raedt R., Leyman L., Verschuere B. (2008). The Karolinska Directed Emotional Faces: A validation study. Cogn. Emot..

[52] Baron-Cohen S., Wheelwright S. (2003). The Friendship Questionnaire: An Investigation of Adults with Asperger Syndrome or High-Functioning Autism, and Normal Sex Differences. J. Autism Dev. Disord..

[53] Baron-Cohen S., Wheelwright S., Hill J., Raste Y., Plumb I. (2001). The “Reading the Mind in the Eyes” Test revised version: A study with normal adults, and adults with Asperger syndrome or high-functioning autism. J. Child Psychol. Psychiatry.

[54] Bryant C. (2018). Researchpy. https://github.com/researchpy/researchpy.

[55] Jones E., Oliphant T., Peterson P. (2001). SciPy: Open Source Scientific Tools for Python. http://www.scipy.org/.

[56] Pedregosa F., Varoquaux G., Gramfort A., Michel V., Thirion B., Grisel O., Blondel M., Prettenhofer P., Weiss R., Dubourg V. (2011). Scikit-learn: Machine learning in Python. J. Mach. Learn. Res..

[57] Seabold S., Perktold J. statsmodels: Econometric and statistical modeling with python. Proceedings of the 9th Python in Science Conference.

[58] Mann H.B., Whitney D.R. (1947). On a Test of Whether one of Two Random Variables is Stochastically Larger than the Other. Ann. Math. Stat..

[59] Groen Y., Fuermaier A.B.M., Den Heijer A.E., Tucha O., Althaus M. (2015). The Empathy and Systemizing Quotient: The Psychometric Properties of the Dutch Version and a Review of the Cross-Cultural Stability. J. Autism Dev. Disord..

[60] Dapretto M., Davies M.S., Pfeifer J.H., Scott A.A., Sigman M., Bookheimer S.Y., Iacoboni M. (2006). Understanding emotions in others: Mirror neuron dysfunction in children with autism spectrum disorders. Nat. Neurosci..

[61] Tassinary L.G., Cacioppo J.T. (1992). Unobservable Facial Actions and Emotion. Psychol. Sci..

[62] Weyers P., Mühlberger A., Hefele C., Pauli P. (2006). Electromyographic responses to static and dynamic avatar emotional facial expressions. Psychophysiology.

[63] Rymarczyk K., Biele C., Grabowska A., Majczynski H. (2011). EMG activity in response to static and dynamic facial expressions. Int. J. Psychophysiol..

[64] Cohen J. (1992). A power primer. Psychol. Bull..

[65] Holland A.C., O’Connell G., Dziobek I. (2021). Facial mimicry, empathy, and emotion recognition: A meta-analysis of correlations. Cogn. Emot..

